# Evaluating the efficacy of the anticancer drug cetuximab by atomic force microscopy†

**DOI:** 10.1039/c8ra03215g

**Published:** 2018-06-13

**Authors:** Qingrong Zhang, Yan Shi, Haijiao Xu, Lulu Zhou, Jing Gao, Junguang Jiang, Mingjun Cai, Yuping Shan

**Affiliations:** School of Chemistry and Life Science, Advanced Institute of Materials Science, Changchun University of Technology Changchun 130012 China shanyp@ciac.ac.cn; Changchun Institute of Applied Chemistry, State Key Laboratory of Electroanalytical Chemistry Chinese Academy of Science Renmin St 5625 Changchun Jilin130022 China caimingjun@ciac.ac.cn; University of Chinese Academy of Sciences Beijing 100049 China

## Abstract

Cetuximab is a monoclonal antibody that binds to the epidermal growth factor receptor, which is important in the growth of many cancers. However, the biophysical characteristics of cetuximab as an anti-cancer drug remain elusive. In this study, we adopted atomic force microscopy to measure the mechanical properties of cancer cells following cetuximab treatment and the biomechanical properties of cetuximab and epidermal growth factor receptor interactions. Atomic force microscopy can be implemented as a platform for further investigations that target the cellular stiffness and affinity of ligand–receptor as a therapeutic choice.

## Abstract 2

Atomic force microscopy can be implemented as a platform for further investigations that target the cellular stiffness and affinity of ligand–receptor as a therapeutic choice.

## Graphical abstract

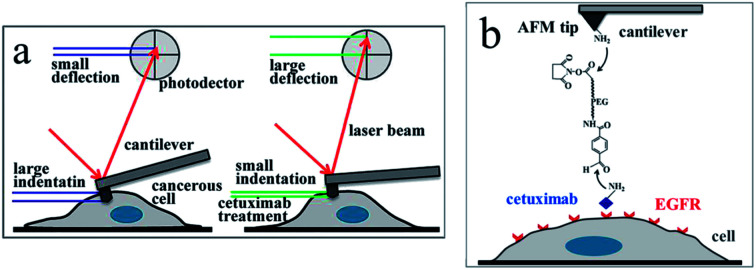

## 1 Introduction

Epidermal growth factor receptor (EGFR), which belongs to the family of trans-membrane receptor tyrosine kinases, and its ligand epidermal growth factor (EGF) are found in the majority of eukaryote cells and play important roles in cellular growth, survival, adhesion, migration, and differentiation.^1^ The abnormal regulation of EGFR is linked with diverse types of cancers,^2^ and monoclonal antibodies (mAbs) specifically targeting EGFR have been developed as therapeutic strategies to achieve tumor regression.^3^ Cetuximab, a chimeric mAb, specifically binds to EGFR with high affinity, blocking growth-factor binding, receptor activation, and subsequent signal-transduction events.^4–7^ The potential of cetuximab to combine with other chemotherapy drugs and increase efficacy without significantly increasing toxicity represents a novel strategy in the treatment of lung cancer and various other types of cancers.^8,9^

Fluorescent imaging and biochemical studies have shown that cetuximab can prevent ligand binding to EGFR by inhibiting receptor internalization.^10,11^ Cetuximab also inhibits the proliferation of various types of cancer cells, thus exhibiting abnormal regulation of EGFR,^12,13^ and clinical studies have shown that cetuximab can significantly inhibit tumor migration.^14^ Several studies have examined the biological and biochemical properties of cetuximab for targeting EGFR to achieve tumor regression *in vitro.* However, due to the limitations in the current methodologies, the effects of cetuximab on the mechanical properties of living cells and the biomechanical properties of cetuximab bound to EGFR on the surface of living cells under physiological conditions remain unclear.

Atomic force microscopy (AFM) is a versatile technique for probing the mechanical properties of living cells and the biomechanical properties of interactions between biomolecules and their receptors on the surface of living cells with nanoscale spatial resolution.^15^ In particular, AFM-based nano-indentation is well adapted to the study of mechanical properties of living cells, owing to its piconewton force sensitivity, sub-nanometer spatial resolution in the vertical direction, and nanometer spatial resolution in the lateral direction. Nano-indentation can therefore be used to probe the mechanical properties of living cells in real time.^16–19^ Single molecule force spectroscopy (SMFS) using AFM is an important tool for studying the forces of interaction between or within the biomolecules, and it can be used to quantify the forces between the ligands conjugated on the AFM tip and bound receptors at the piconewton level.^20–23^ Interaction forces and binding kinetics of ligand–receptor, antibody–antigen, and other systems have been widely investigated *in situ* using SMFS.^24–26^ Herein, we used AFM to characterize the changes in mechanical properties of living cells following cetuximab treatment and to explore the single molecular interaction forces between cetuximab conjugated on the AFM tip and EGFR on the surface of living cells.

## 2 Experiments

### 2.1 Cell culture

The A549 lung cancer cells were provided by the Stem Cell Bank, Chinese Academy of Sciences (Shanghai, China). The cells were maintained in Dulbecco's Modified Eagle's Medium (DMEM; HyClone) supplemented with 10% fetal bovine serum (FBS), 100 U ml^−1^ penicillin, and 200 μg ml^−1^ streptomycin (Biological Industries, Israel) under a humidified atmosphere of 5% CO_2_ at 37 °C. Prior to experiments, the cells were cultured in 35 mm Petri dishes at a final cell density of approximately 5 × 10^4^ cells for 24–36 h and then rinsed three times using 1 ml phosphate buffer saline (PBS; 137 mM NaCl, 2 mM KCl, 8 mM Na_2_HPO_4_, 1.5 mM KH_2_PO_4_, pH 7.4).

### 2.2 Measurement of single cell stiffness

An Agilent 5500 AFM instrument (Agilent Technologies, Chandler, AZ) was used to obtain force–distance curves directly from cells cultured in Petri dishes, as previously described.^27^ A tip with a triangular cantilever (*k* = 0.036 N m^−1^) was used to precisely apply a compression force orthogonal to the cell, where measurements are less affected by the stiffness of the substrate. To ensure the veracity of elastic stiffness, force–distance curves were acquired from the substrate for the calibration of the cantilever deflection signal. The number of cells tested in each condition ranged from 4 to 6, with over 530 force–displacement curves generated per condition.

### 2.3 Young's modulus calculation

Young's modulus of cells was calculated from the force–distance curves using the Hertz model.^28^ The model hypothesized that the indented area was continuous and incompressible at small deformations. Although this model lacks very high precision, it has been shown to be adequate for estimating cell elasticity and is therefore well established for this purpose.^29,30^ The mechanical properties of living cells can vary within the same protocol and model, although the data obtained are considered reliable for comparison purposes.^30^ The Young's modulus was calculated according to the following eqn (1):1
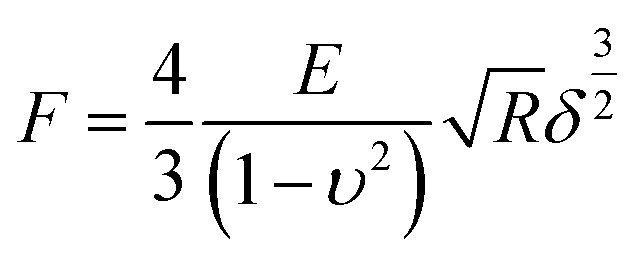
here, *F* is the loading force, *E* is the Young's modulus, *R* is the tip radius, and *δ* is the half-opening angle of the sharp tip. The cells were considered to be linearly elastic, isotropic, and incompressible at smaller strain values, as a Poisson ratio of 0.5 was used. Young's modulus was obtained by the AtomicJ software (Poland).^31^

### 2.4 Tip functionalization

Cetuximab (EFEBIO, Shanghai, China) was functionalized onto AFM tips (MSCT, Si_3_N_4_, Bruker Probe) as previously described.^32^ In brief, AFM tips were cleaned in a UV cleaner, immediately transferred to a desiccator and incubated with 50 μl 3-aminopropyltriethoxysilane (APTES) and 20 μl triethylamine using a vapor phase deposition method for 2 h. A 12 nm NHS-PEG27-acetal cross-linker (Acetal-PEG27-NHS, FW∼1598, Institute of Biophysics, Johannes Kepler University, Austria) was attached to the AFM tips modified with APTES by incubating the tips for 2 h with 6.6 mg ml^−1^ of PEG linker in chloroform containing 1% (v/v) of triethylamine. Next, the AFM tips modified with PEG underwent acetal cleavage following 10 min of treatment in 1% citric acid. After washing three times with PBS, the tips were immersed in a mixture of 100 μl cetuximab solution in PBS and 4 μl of 1 M NaCNBH_3_. After functionalization for 60 min, 10 μl of 1 M ethanolamine was added to the solution to passivate the unreacted aldehyde groups. The AFM tips were subsequently washed three times with PBS and stored at 4 °C until use.^33^ EGF (Sigma-Aldrich, Shanghai, China) was attached to AFM tips according to the same process as that used for cetuximab.

### 2.5 Binding force measurements

Force measurements were determined using an Agilent 5500 AFM instrument (Agilent Technologies, America). All single molecule force spectroscopy experiments were performed in PBS buffer at 37 °C in the contact mode.^34,35^ For each experiment, 3000–10 000 original force curves were obtained from 4–10 cells. The unbinding force between ligand and receptor was calculated from the withdrawal region of the force–distance curve with a user-defined program in Matlab. By averaging the values of binding forces obtained from three independent experiments,^36^ the most probable molecular interaction force was determined.

## 3 Results and discussion

The mechanical properties of normal cells upon their transformation into tumor cells are abruptly altered, accompanied by a decrease in the cell elasticity.^37–39^ Given the favorable effects of cetuximab on tumor regression,^8^ we postulated that it might exert an effect on the mechanical properties of cancer cells. To support this hypothesis, we adopted nano-indentation to determine the *in situ* mechanical properties of cancer cells following cetuximab treatment.

**Fig. 1:**
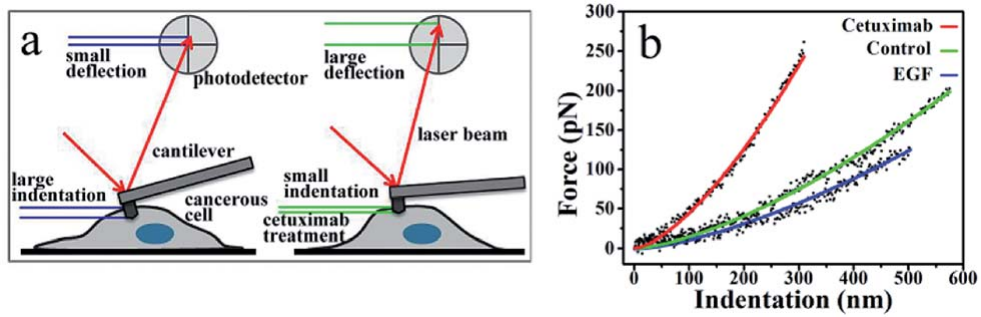
(a) Detection of indentation based on AFM measurements. Cancer cells show higher ability to deform (*i.e.*, greater indentation depth); therefore, cantilever deflection is reduced. Following cetuximab treatment, the indentation depth is reduced because of increased cell stiffness, resulting in increased deflection of the probing cantilever. (b) Force-*versus*-indentation curves in A549 cells following cetuximab treatment (red), control (green), and with EGF treatment (blue). Lines denote a smooth fit to experimental data (dots).

An AFM probing tip mounted at the end of a micro cantilever was used to indent the cell, resulting in an automatic deflection of the cantilever. The deflection was detected using a photoelectric detection system. A photodiode, with its active area sectored into four quadrants, was used to record the laser beam reflected from the end of the cantilever (Fig. 1a). The cantilever deflection was plotted to determine the cancer cell indentation depth following cetuximab treatment, which was found to be significantly lower than that of unmodified cancer cells (Fig. 1a and b). A typical force–indentation curve is shown in Fig. 1b. Following cetuximab treatment, a steep force–indentation curve (red) was observed, indicating a significant increase in cell stiffness compared with that of an untreated cell (green) under the same force. EGF treatment induced cancer cell proliferation, which was indicated by a more gradual force–indentation curve (blue), reflecting a reduction in stiffness compared with that in the control group (green) (Fig. 1b).

**Fig. 2:**
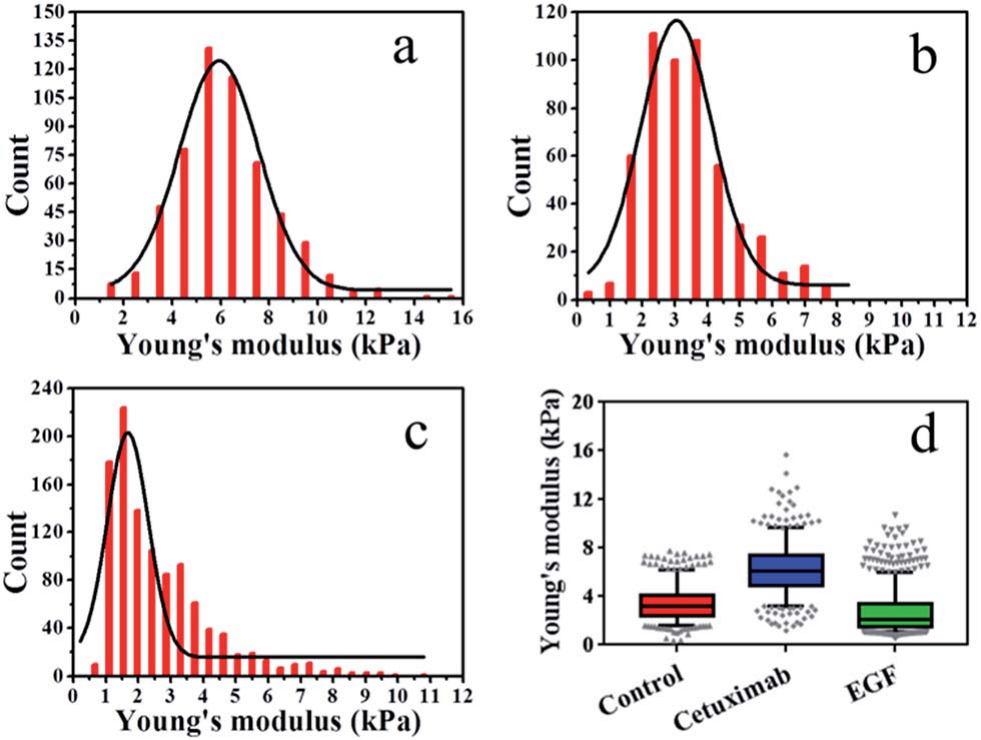
Young's modulus distributions with Gaussian fit functions for living cells. (a) Cetuximab treatment, (b) control, (c) EGF treatment. (d) Box plots of modulus values for control, cetuximab, and EGF. Boxes represent the data distribution (50%), with vertical lines through the boxes representing the distribution of 95% of all data and horizontal lines in every box representing the average values. The symbols at the top and bottom of the plot represent the staggered distribution of outliers.

The elasticity (Young's modulus, *E*) of individual cells was calculated from the approach stage of the force–distance curves obtained at 37 °C under a rate of 2 μm s^−1^. The Young's modulus of cancer cells under different conditions is displayed in Fig. 2. In each experiment, 500–1000 original force curves were obtained from 4–6 cells, and Young's modulus (mean ± S.D.) was obtained from the Gaussian fitting of Young's modulus distribution. Prior to force–distance testing, A549 lung cancer cells were pre-incubated with cetuximab (final concentration, 20 nM)^40^ in DMEM at 37 °C for 12 h. The Young's modulus value was approximately 6.22 ± 2.0 kPa at a loading velocity of 2 μm s^−1^, as illustrated in Fig. 2a, and this value was significantly higher than that of the control group (3.42 ± 1.4 kPa; Fig. 2b). The data showed that the rigidity of cancer cells following cetuximab treatment was increased compared with that observed for the control group (Fig. 2d). This increase in rigidity correlated with reduced migration and proliferation of cancer cells in the treatment group. To verify that the cell stiffness was induced by cetuximab, the cell stiffness was obtained on triple-negative breast cancer cell lines and triple-negative breast cancer cells of soft substrates. The results indicated that cetuximab can also induce membrane stiffening in triple-negative breast cancer cell lines and triple-negative breast cancer cells of soft substrates (Fig. S1 and S2†).

In addition, we treated A549 cells that were incubated with EGF (final concentration, 1 ng ml^−1^)^41–43^ in DMEM at 37 °C for 12 h, and we found that the Young's modulus value was approximately 2.65 ± 1.6 kPa at a loading velocity of 2 μm s^−1^, as shown in Fig. 2c. Following EGF treatment, the value for the Young's modulus decreased from 3.42 ± 1.4 kPa to 2.65 ± 1.6 kPa compared with that of the control (Fig. 2d). Overstimulation with EGF can lead to decreased cancer cell rigidity because the migration and proliferation of cancer cells are induced by EGF.^3,4^ Taken together, these results suggested that cetuximab can significantly decrease the migration and proliferation of lung cancer cells by enhancing their rigidity while efficiently achieving tumor regression.

Furthermore, SMFS was used to evaluate the interaction force between EGFR on the cell membrane and cetuximab or EGF. Cetuximab was covalently conjugated onto an AFM tip *via* a heterobifunctional aldehyde-PEG (poly(ethylene glycol))-NHS linker.

**Fig. 3:**
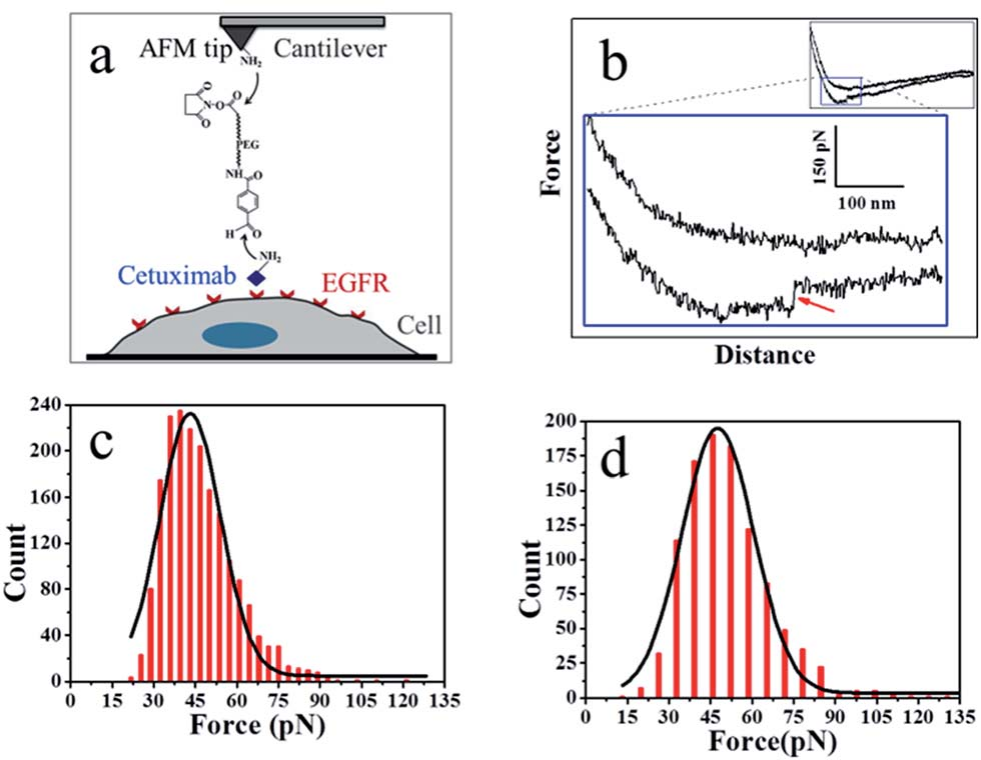
(a) The experimental procedure. The heterobifunctional aldehyde-PEG (poly(ethylene glycol))-NHS cross-linker is covalently bound to the APTES-attached AFM tip through the NHS-ester. The cetuximab is conjugated to the aldehyde. (b) A typical force–distance cycle of the specific interaction between cetuximab and EGFR. The lower panel represents amplified regions of the entire profile, showing a force signal of the integral force–distance cycle events (inset). (c) Histogram of cetuximab–EGFR binding forces. (d) Histogram of EGF–EGFR binding forces (*n* > 1000).

As shown in Fig. 3a, the PEG linker was immobilized on the aminated AFM probe through the NHS ester terminus, and the amino groups of the side chain or N-terminal of cetuximab reacted with the benzaldehyde moiety of the immobilized linker. Cetuximab molecules attached to the AFM tip were allowed to specifically bind to EGFR molecules on cell membranes.

Fig. 3b shows a typical force–distance curve of cetuximab interacting with EGFR on the living cell membrane. The force–distance curve begins from the right side of the upper line, which represents the approach of the force–distance cycle; the lower line shows the retrace process. The force–distance curve of the full profile is shown in the inset of Fig. 3b. As the AFM tip approached and then interacted with the cell membrane, a gradual slope appeared as a result of the deformation of cell membranes from the tip pressing on the living cells; such a slope is a feature of force–distance curves for soft surfaces or living cells.^25^ The cetuximab–EGFR complex was formed during the approach period. When the AFM tip was withdrawn from the cell surface, the specific interaction between EGFR on the cell and cetuximab modified on the tip was ruptured, causing a force signal to be detected (Fig. 3b, red arrow). The dual rupture events were also detected in the dissociation of cetuximab–EGFR (Fig. S3†).

The binding force of cetuximab–EGFR was measured using the force–distance curves of rupture events. The interaction force between cetuximab and EGFR on A549 cells was in the range of 22–120 pN, with the most probable value at 46.68 ± 12.9 pN at a retraction velocity of 1.96 μm s^−1^, as illustrated in Fig. 3c. The interaction force between EFG and EGFR on A459 cells was also detected, as shown in Fig. 3d. The unbinding forces ranged from 21 to 132 pN, with the most probable distribution at 50.99 ± 15.6 pN at a retraction velocity of 1.96 μm s^−1^.

**Fig. 4:**
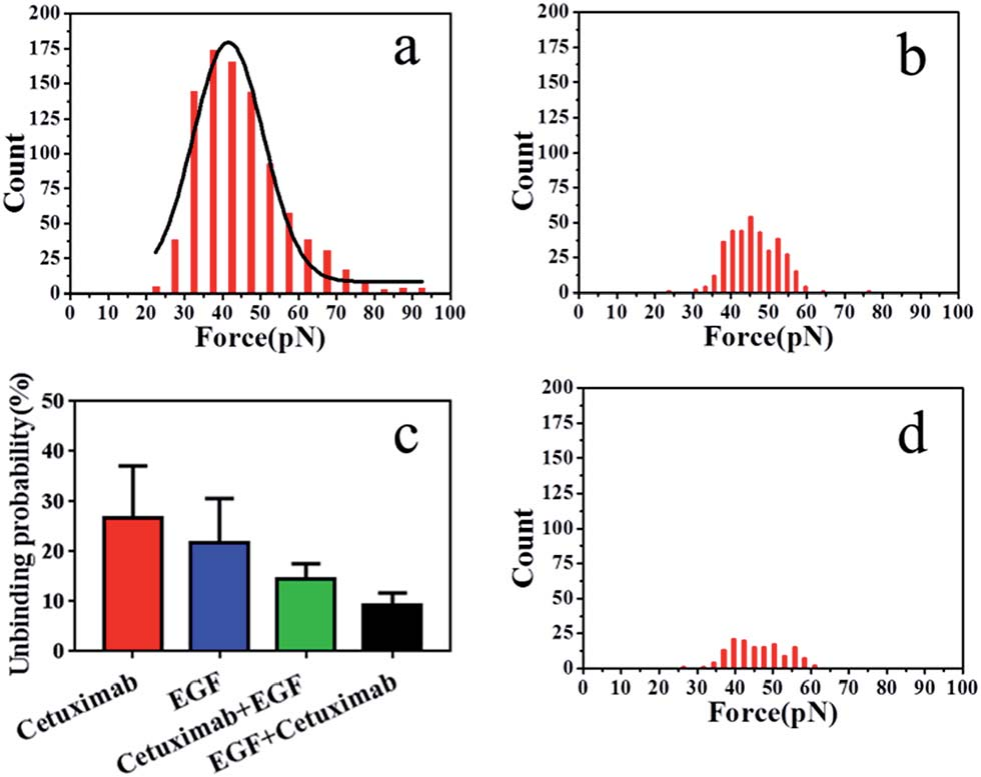
(a) Histogram of cetuximab–EGFR binding forces in the presence of EGF and (b) of EGF–EGFR binding forces in the presence of cetuximab. (c) Average binding probabilities of various ligands with EGFR under different conditions. Binding probabilities of cetuximab-modified AFM tips with EGFR (red), EGF-modified AFM tips with EGFR (blue), cetuximab-modified AFM tips in the presence of free EGF (green), and EGF-modified AFM tips in the presence of free cetuximab with EGFR (black). (d) Histogram of interaction forces of PEG-modified AFM tips with the membrane of A549 cells.

The binding probability was also calculated from the number of force curves with rupture events divided by the overall number of the force curves. The binding probability of EGF–EGFR decreased from 26.9 ± 3.24% to 21.8 ± 3.60% compared with that of cetuximab–EGFR, as shown in Fig. 4c. The specific unbinding events of EGF–EGFR were reduced. Cetuximab–EGFR was found to have a relatively higher binding probability but a virtually identical interaction force to that of EGF–EGFR. Therefore, we proposed that cetuximab had a stronger binding efficacy to EGFR than EGF.

To further verify the affinity of cetuximab for EGFR, we evaluated the interaction probability of cetuximab–EGFR in the presence of free EGF (final concentration, 1 ng ml^−1^). The binding probability decreased from 26.9 ± 3.24% to 14.6 ± 1.25% (Fig. 4c). The histogram of unbinding forces is depicted in Fig. 4a. The unbinding forces were in the range of 22–93 pN, and the most probable unbinding force was 44.87 ± 11.6 pN. Subsequently, the EGF–EGFR interaction was detected in the presence of free cetuximab (final concentration, 20 nM). The binding probability reduced from 21.8 ± 3.60% to 9.2 ± 1.04%, as illustrated in Fig. 4c, with an unbinding force of 45.97 ± 0.3 pN, as shown in Fig. 4b. Before and after blocking with free competitors, the binding probability of cetuximab to EGFR was higher than that of EGF, despite the similar binding force value. These results further indicated that cetuximab bound more strongly to EGFR than EGF. Cetuximab can prevent EGFR dimerization and subsequent activation by EGF, thus triggering apoptosis in cancer cells.^1,5,44^

To verify that the interaction forces observed were indeed a consequence of the specific cetuximab–EGFR interaction, negative control experiments were performed. We found that most of the force signals disappeared when the AFM tip was tethered with PEG linker, as shown in Fig. 4d. For the testing with the bare tip, the interaction force disappeared, as shown in Fig. S4.† Control experiments indicated that the binding interactions between EGFR on A549 cells and ligands on tips were specifically and efficiently detected.

The effects of mAbs drugs have been widely investigated using chemical, biomedical, pharmacological, and clinical methods; all of these methods require sample pre-processing owing to the complexity of detected drug efficacy. We used nano-indentation combined with AFM-based SMFS to assess the efficiency of cetuximab in cancer therapy. The stiffness of cancer cells following treatment with cetuximab was significantly higher than that of normal cells under the same conditions. This finding was in accordance with the increased stiffness of normal cells compared with that of the cancer cells.^39^ Nanomechanical analysis is a robust method for measuring the effects of anticancer drugs on living cells under physiological conditions. In addition, the results of SMFS indicated that the binding ability of cetuximab to EGFR in single cells was stronger than that of EGF to EGFR. This finding was in accordance with the result that the affinity of cetuximab with respect to EGFR interaction was higher than that for the interaction of EGF with EGFR.^40^

## 4 Conclusions

These results indicate that AFM can effectively evaluate the efficacy of cetuximab with respect to its mechanical and biomechanical properties under physiological conditions. Our methodology represents a potential strategy to examine the functions of designed antibodies for cancer treatment *in vitro*.

## Conflicts of interest

There are no conflicts to declare.

## Supplementary Material

RA-008-C8RA03215G-s001
