# Supplementary material for: Evaluating the efficacy of the anticancer drug cetuximab by atomic force microscopy
Source: RSC Adv. 2018 Jun 13;8(39):21793–7. doi: 10.1039/c8ra03215g (PMC9081852; doi:10.1039/c8ra03215g)
Supplement: RA-008-C8RA03215G-s001 [file RA-008-C8RA03215G-s001.pdf]

Electronic Supplementary Material (ESI) for RSC Advances.
This journal is © The Royal Society of Chemistry 2018

Supporting information

The Young's modulus of MDA-MD-231 were approximately 4.7 ± 0.12 kPa (n=499), a value significantly higher than that of the control group without treated cetuximab (2.6 ± 0.09; n=344; Fig. S1). The results indicate that cetuximab can induces membrane stiffening in breast cancer cell lines.

The MDA-MD-231, triple-negative breast cancer cells, was kindly provided by the National Infrastructure of Cell Line Resource (Beijing, China). MDA-MD-231 cells were maintained in RPMI Medium (RPMI; HyClone) supplemented with 10% fetal bovine serum (FBS), 100 U/ml penicillin, and 200 μg/ml streptomycin (Biological Industries, Israel) under a humidified atmosphere of 5% CO^2 at 37 °C. MDA-MD-231 cells were cultured in 35 mm Petri dishes at a final cell density of approximately 5×10^4 cells for 24–36 h. MDA-MD-231 was pre-incubated with cetuximab (final concentration, 20 nM) in RPMI at 37 °C for 12 h.

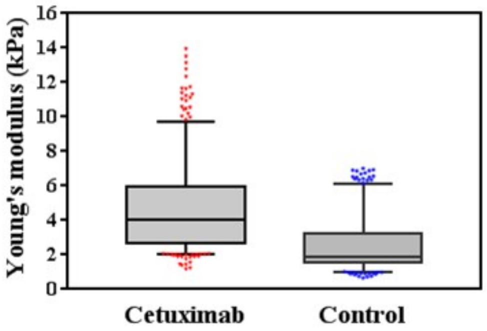

Fig. S1. Box plots of Young's modulus values for cetuximab and control. Boxes represent the data distribution (50%), with vertical lines through the boxes representing the distribution of 95% of all data and horizontal lines in every box representing the average values. The symbols at the top and bottom of the plot represent the staggered distribution of outliers.

The Young's modulus values were approximately 4.1 ± 0.07 kPa (n= 652), a value higher than that of the control group without treated cetuximab (2.2 ± 0.05 kPa; n=454; Fig. S2.) in soft substrate. The data showed that the rigidity of cancer cells was gently decreased in soft substrate, but cetuximab can distinctly induces stiffening of cancer cell in soft substrate.

The Parafilm (Sigma) were pressed on a 35 mm polystyrene petri dish for better adhesion. The MDA-MD-231 cells were cultured in the parafilm-covered petri dish at a final cell density of approximately 5×10^4 cells for 24–36 h. The MDA-MD-231 was pre-incubated with cetuximab (final concentration, 20 nM) in RPMI at 37 °C for 12 h.

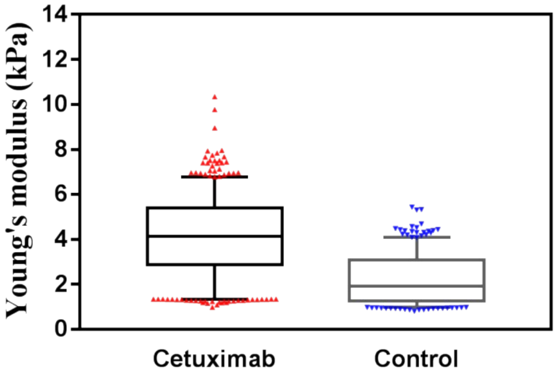

Fig. S2. Box plots of Young's modulus values for cetuximab and control in soft substrate. Boxes represent the data distribution (50%), with vertical lines through the boxes representing the distribution of 95% of all data and horizontal lines in every box representing the average values. The symbols at the top and bottom of the plot represent the staggered distribution of outliers.

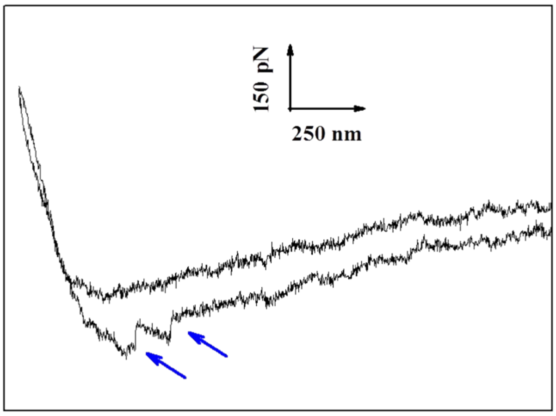

Fig. S3 The force-distance curve of the dual rupture event of cetuximab-EGFR.

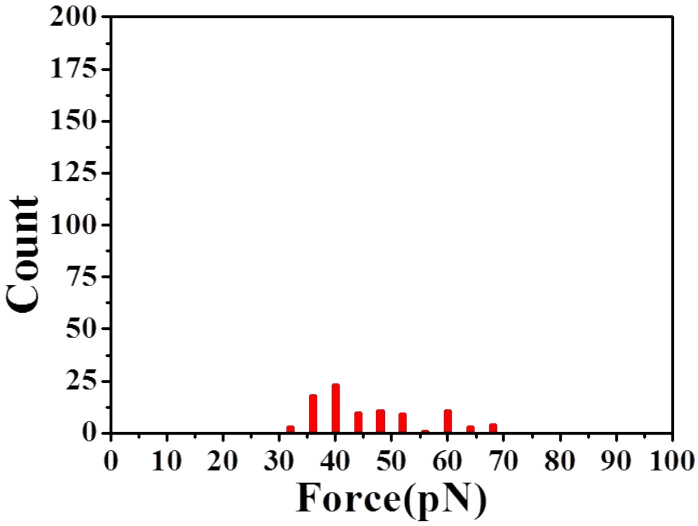

Fig. S4 Histogram of binding forces on bare tips.
